# Community-based rehabilitation: Working in partnership with eye care

**Published:** 2013

**Authors:** Joerg Weber

**Affiliations:** Research fellow: International Centre for Evidence in Disability, London School of Hygiene and Tropical Medicine, UK.

**Figure:**
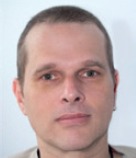
Joerg Weber

Community-based rehabilitation (CBR) was introduced in the late 1970s as a new approach to providing rehabilitation services at community level.

Over the years, the concept of CBR has widened to include much more than just medical rehabilitation. These days, organisations providing CBR aim to empower people with disabilities to enjoy education, health, and wellbeing, and to participate fully in social, cultural, civil, and political life. They do this by offering support to people with disabilities and by supporting projects – such as income-generating projects – in which people with disabilities can take the lead. This stimulates local initiatives and encourages self-determination.

**The benefits of linking eye care workers and CBR programmes**

Any response to the needs of people with visual impairment and their families will be more effective if eye care workers and CBR programme staff can work together at the community level.

Eye care services play an important role in identifying people with visual impairment and addressing their initial requirements, including treatment. However, many people with visual impairment may never come to the eye clinic! They may face a range of different barriers, including physical, economic, and attitudinal barriers. For example, they may be unaware of what is available, they may believe that they cannot afford eye care, or perhaps physically getting to the clinic is difficult for them.

To improve the coverage of eye services, eye health workers can train CBR workers to refer or even undertake basic screening in the communities they regularly visit. This way, eye care services will be provided to a greater number of people. CBR workers should also attend clinics held by the eye health services. This way they can better ensure that necessary follow-up visits or treatments take place, and that the family of the patient remains informed and motivated about medical or rehabilitation procedures.

**The importance of CBR referrals**

Eye care workers can refer people with low vison or other visual impairments to a CBR programme. This is very important in order to assist a person with visual impairment to retain good levels of function and independence.

Because they can utilise their networks and take a holistic approach to patient care, CBR projects are efficient hubs for obtaining referrals and other support or action on behalf of patients and their carers. This can help people with visual impairments to overcome a multitude of barriers to inclusion in the community at all levels in society, and at different stages in life.

CBR can support eye care delivery in the areas of health, education, livelihood, society, and empowerment. The following are possible further steps for each of these at community level.

**Actions for health**

- Promote eye health by creating awareness (how to recognise signs of visual impairment, the need for early detection, information about low vision aids, medical procedures, etc.).
- Refer people with suspected visual impairment for further testing and follow-up examinations.
- Advocate for appropriate low vision services at health centres and hospitals as close to the community as possible.
- Inform children and adults with visual impairment, their family members, and the general public about available options for the inclusion of people with visual impairment in the community.

**Actions for education**

- Facilitate educational opportunities for children and students with visual impairment at all pre-primary, primary, secondary and higher levels of education.
- Educate teachers in regular schools about the impact of visual impairment and the provision of an effective learning environment for children with visual impairment. Encourage the inclusion of these topics in teacher education programmes.
- Ensure that families of children with visual impairment understand the practical implications of local policies relating to the education of such children.
- Recruit and train people with visual impairment as CBR workers and teaching assistants.

**Actions for livelihood**

- Ensure that people with visual impairment and their families are fully aware of their rights with respect to employment.
- Ensure that people with visual impairment have access to social protection mechanisms such as social security.
- Advocate among trade unions and employers to promote inclusion of people with visual impairment.

**Actions for society**

- Help to reduce stigmatisation of people with visual impairment.
- Raise awareness in the community of the causes and nature of, and possible solutions, for visual impairment.
- Help to ensure that people with visual impairment have access to all cultural, recreational, sport, religious, and other activities in the community.

**Actions for empowerment**

- Support the establishment of local support and self-help groups for people with visual impairment
- Hire and train people with visual impairment as CBR workers and in primary health care.

Two-way referrals and close networking between eye care and CBR will significantly improve medical, educational, econonic and social outcomes for people with visual impairment and their families.

**Figure:**
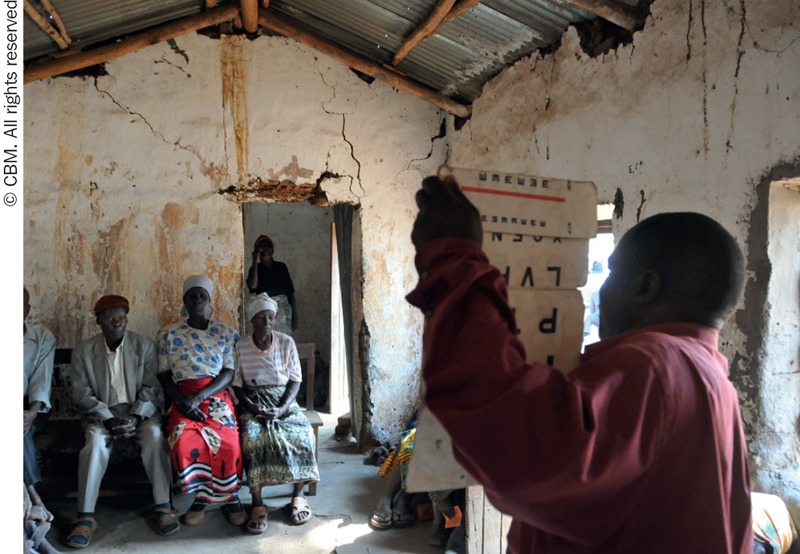
Fieldworkers conducting simple vision tests, showing how CBR can support detection and referral. MALAWI

